# From Our Foreign Correspondent

**Published:** 1983-10

**Authors:** 


### From our Foreign Correspondent

### Abu Dhabi

The Emirate of Abu Dhabi is undoubtedly a creation of man: there has been little obvious in the way of divine intervention. Even the desert, for the most part, lacks style as it skirts the Arabian Gulf. To call it lunar does a disservice to the moon. Along the road leading westwards from the city of Abu Dhabi itself the landscape is further disfigured (yes, it is possible) by detritus derived from automobiles and trucks – tyres, wheels and whole or part of vehicles abandoned in ugly profusion.

It is of course oil which has given this part of the world the importance which it merits on no other grounds and the one million inhabitants of the Emirates as a whole are outnumbered at least four to one by newcomers drawn towards the wealth generated by the black gold. Many of these are fellow Arabs and the most significant group of these are the talented and industrious Palestinians who occupy most of the posts of any importance in government and industry. Another and very large group comes from the Indian subcontinent and many of these are illegal immigrants. In this feudal state they are the serfs, discriminated against and exploited.

The local, still nomadic, bedouin are a proud people whose way of life is gradually being suborned by the vulgar wealth in which they share. During a recent visit there to help commission a small hospital in the desert, I was faced with a local bedu who wished investigation and treatment for an imaginary illness; when I refused, my interpreter, a Lebanese male nurse, stood between me and the torrent of abuse which, loosely translated, questioned my veracity and my parentage. On a previous occasion when an effendi had refused to treat one of the faithful under similar circumstances a gift to the hospital of two ambulances was immediately cancelled by the local sheikh.

Another big problem was the animosity towards male doctors in examining female patients although nowhere does the Koran forbid it. The greatest hostility came from the husbands who belonged to a minority Palestinian fundamental Muslim group. The quality of the doctor and the care provided seemed to be of secondary importance.

The centrepiece of the small hospital was a beautiful marble hall surrounded by a profusion of potted plants flown in from Spain at a cost of over £100,000. This highlights one other lasting impression of the Middle East – appearances matter, or, to put it another way, it is not how things *are* which is important but rather how they seem to be.

Gordon M. Stirrat

